# Three-Dimensional Measurement of Proximal Humerus Fractures Displacement: A Computerized Analysis

**DOI:** 10.3390/jcm12124085

**Published:** 2023-06-16

**Authors:** Thomas Ripoll, Mikaël Chelli, Tyler Johnston, Jean Chaoui, Marc-Olivier Gauci, Heloïse Vasseur, Sergii Poltaretskyi, Pascal Boileau

**Affiliations:** 1 Unité de Recherche Clinique (UR2CA), Université de Nice Côté d’Azur, 06000 Nice, France; thomas.ripoll@hotmail.fr (T.R.); tyler.johnstonmd@gmail.com (T.J.); marcoliviergauci@gmail.com (M.-O.G.); 2 Hôpital Pasteur 2—IULS, 30 Voie Romaine, CÉDEX 1, 06001 Nice, France; vasseurh@hotmail.com; 3 Institut de Chirurgie Réparatrice—Groupe Kantys, 06004 Nice, France; boileau.pascal@wanadoo.fr; 4 Imascap, 29280 Plouzané, France; jean.chaoui@wright.com (J.C.); sergii.poltaretskyi@gmail.com (S.P.)

**Keywords:** proximal humerus fractures, Neer classification, tuberosity displacement, proximal shaft, CT-scan study, 3D planning

## Abstract

Neer’s classification for proximal humerus fractures (PHFs) uses 10 mm and 45° thresholds to distinguish displaced fragments. While this system was originally developed referencing 2D X-rays, fracture displacements occur in three dimensions. Our work aimed to develop a standardized and reliable computerized method for measuring PHF 3D spatial displacements. CT scans of 77 PHFs were analyzed. A statistical shape model (SSM) was used to generate the pre-fracture humerus. This predicted proximal humerus was then used as a “layer” to manually reduce fragments to their native positions and quantify translation and rotation in three dimensions. 3D computerized measurements could be calculated for 96% of fractures and revealed that 47% of PHFs were displaced according to Neer’s criteria. Valgus and varus head rotations in the coronal plane were present in 39% and 45% of cases; these were greater than 45° in 8% of cases and were always associated with axial and sagittal rotations. When compared to 3D measurements, 2D methods underestimated the displacement of tuberosity fragments and did not accurately assess rotational displacements. The use of 3D measurements of fracture displacement is feasible with a computerized method and may help further refine PHF analysis and surgical planning.

## 1. Introduction

Proximal humerus fractures (PHFs) are the third most common fracture in individuals over 65 years of age [1,2]. Neer’s conventional classification system [3,4] is currently the most widely used system to describe these fractures, on account of its simplicity and prognostic value [5]. In this paradigm, Neer describes four principle fragments (humeral head, greater tuberosity, lesser tuberosity, humeral shaft) and categorizes a fragment as displaced if its translation is greater than 10 mm and/or rotation is greater than 45°. In the Neer approach, displacements are primarily due to muscle and tendon forces. This classification system is also of surgical interest and utility as a means to select the most appropriate therapeutic strategy, as restoration of proximal humerus anatomy remains the key to the management of these fractures [6,7]. Currently, however, the use of Neer’s classification system is limited by poor inter- and intra-observer reproducibility [8,9,10]. Analysis of recent literature also shows that the application of Neer’s classification is not standardized [11,12]. While other classification systems have been proposed and utilized, currently no classification system defines a spatial reference frame in which to describe displacements [13,14,15,16,17,18]. Meanwhile, displacements of each fragment can occur in independent planes with associated rotations and translations in three dimensions.

PHF management remains challenging; contributing factors include poor reproducibility of different classification systems [19,20,21,22], lack of consensus on surgical indications [23,24,25], and sequelae/complications that are difficult to treat [26,27]. Specifically, healing of tuberosities in anatomical position is the most important factor known to influence outcomes [6,7,28,29,30,31], and measurement of their displacements is critical to guide surgical indications. 

This study aimed to develop a three-dimensional computerized method to accurately measure the displacements of epiphyseal fracture fragments in PHFs and compare these measurements with those obtained by previously described manual 2D methods.

Our hypothesis was that a statistical shape model of the pre-fracture humerus could be employed to accurately measure three-dimensional translation and rotational displacements of tuberosity and head bone fragments in PHFs.

## 2. Methods

### 2.1. Study Design

**Figure 1:**
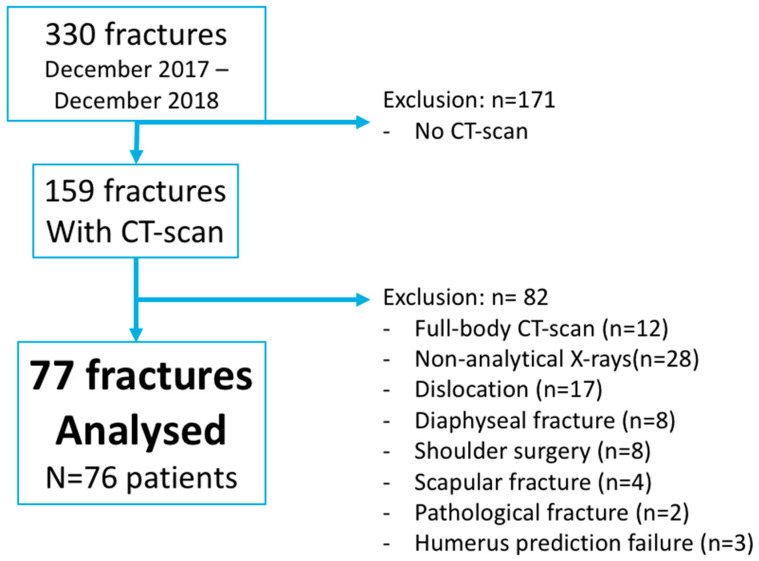
Flowchart.

We conducted a retrospective feasibility study of PHFs admitted to our level 1 trauma center. During a one-year period (between December 2017 and December 2018), all patients older than 18 years who underwent a shoulder CT scan within 48 h of trauma were included. Exclusion criteria are summarized in Figure 1. CT scans of 77 PHFs (in 76 patients) were obtained and analyzed. Epidemiological data are summarized in Table 1. The study was conducted in accordance with the Declaration of Helsinki and the investigation was approved by the hospital’s institutional review board (IRB: IULS-2017-09-FX 3D-RIPOLL).

### 2.2. Image Acquisition

Unilateral shoulder CT scans (including scapula, proximal humerus, and proximal humeral shaft) were performed by the level 1 trauma center radiology department with a General Electric Optima 64-bar device with a helicoidal acquisition, bone fenestration, and 0.625 mm thick slices. The protocol used a kilovoltage peak of 120 kVp, while milliamperage was calculated automatically according to scout imaging (between 100 and 500 mA), and a BONEPLUS convolution filter was used (Kernel).

### 2.3. Manual Analysis

Independent analysis of fracture displacements on X-rays as well as 2D and 3D CT reconstructions was performed by three observers: an orthopedic surgeon specializing in shoulder surgery, an orthopedic surgery resident, and a radiologist specializing in osteoarticular imaging. 

For each fracture, the following variables were evaluated by each observer: the number of displaced fragments according to Neer’s conventional criteria (≥10 mm displacement or ≥45° of angulation), displacement of tuberosities and diaphysis (grouped in 3 ranges: <5 mm; 5–9.9 mm; ≥10 mm), and humeral head rotation (grouped in 5 classes: neutral, varus < 45°, varus ≥ 45°, valgus < 45°, valgus ≥ 45°).

### 2.4. Computerized Analysis of Fracture Displacements

A computerized method to standardize the measurement of epiphyseal fracture fragment displacement was developed. This method was based on the prediction of the patient-specific pre-fracture proximal humerus, which was then used as a blueprint layer onto which displaced fragments were manually reduced in three dimensions. Each of the 6 steps is further detailed below.

1. Segmentation

**Figure 2:**
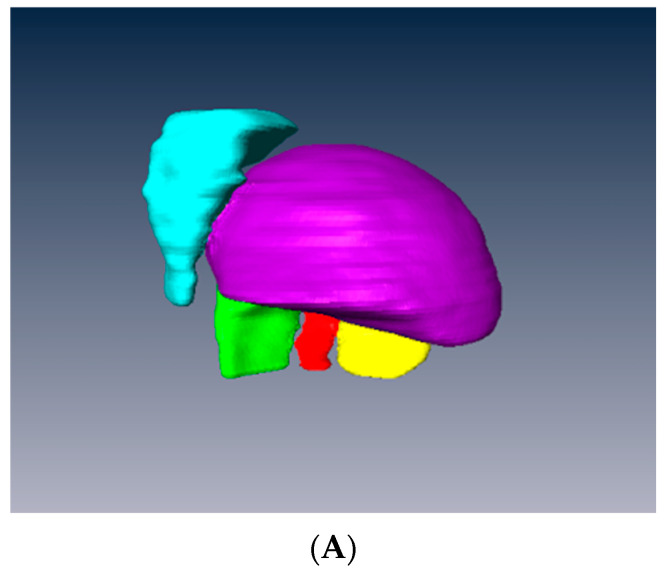

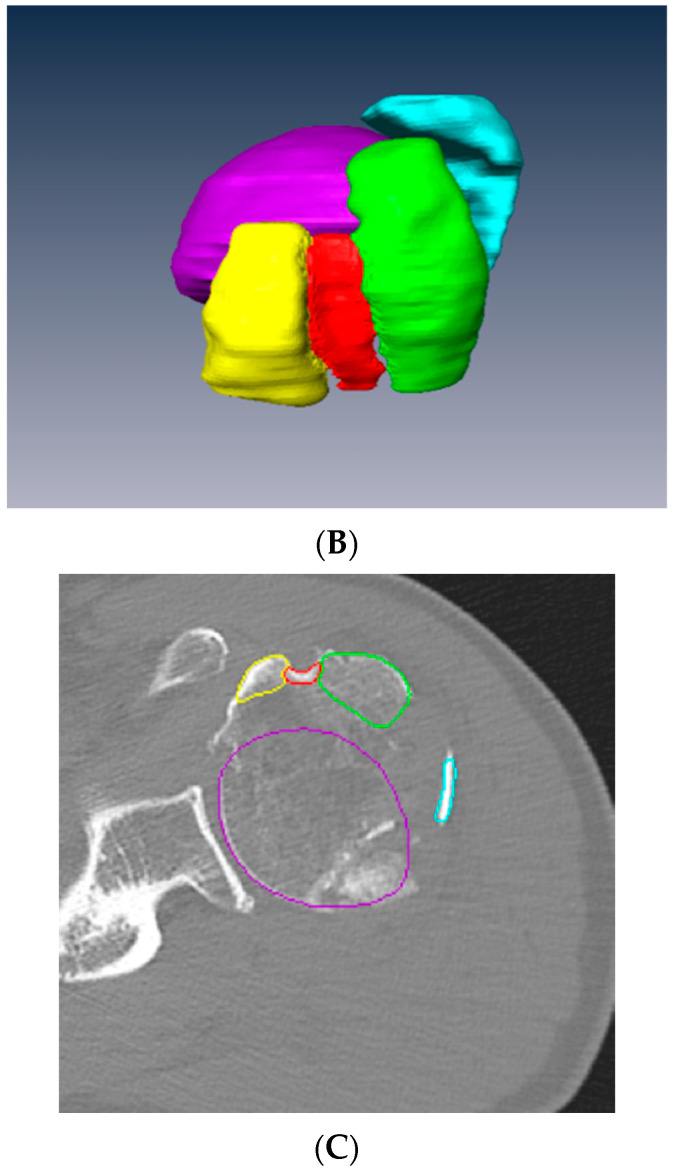
Segmentation of fractured fragments using AMIRA^®^ software. (**A**) 3D mesh posterior view; (**B**) 3D mesh anterior view; (**C**) Fragment boundaries were contoured by hand on every other axial CT section. Here, the humeral head is in purple, the greater tuberosity in green (unfractured) and blue (posterior fractured portion), and the lesser tuberosity in yellow.

The objective of segmentation was to obtain a three-dimensional model (3D mesh) (Figure 2A,B) of each fractured bone fragment using AMIRA^®^ software (version 5.3, Zuse Institute Berlin, Berlin, Germany). On axial CT scan sequences, each fragment was manually outlined based on cortical bone contours: greater tuberosity (GT), lesser tuberosity (LT), and humeral head (Figure 2C). Diaphysis and scapula were automatically segmented by Glenosys software (BluePrint) (version 10.4.4, IMASCAP S.A.S, Plouzane, France).

2. Proximal humerus pre-fracture anatomy prediction

**Figure 3:**
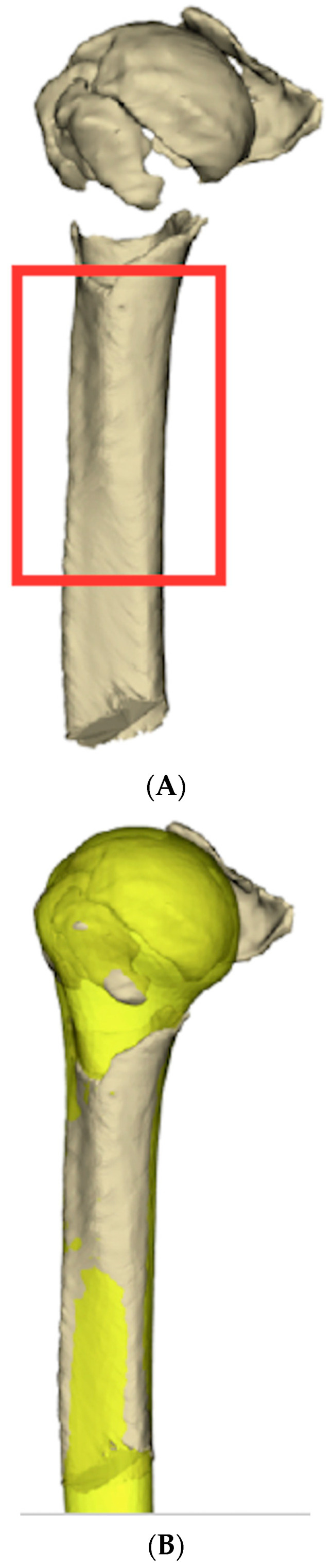
Pre-fractured humerus prediction. (**A**) A statistical shape model was used to reconstruct the pre-fracture proximal humerus from the most proximal 6 cm of unfractured shaft; (**B**) Predicted pre-fracture proximal humerus displayed in yellow. The original unfractured shaft is displayed in beige.

Based on the work of Poltaretskyi et al. [32], pre-fracture proximal humerus anatomy was extrapolated by building up from the most proximal 6 cm of the unfractured shaft (Figure 3A,B). This technique was based on a Statistical Shape Model (SSM) that predicted the pre-morbid morphology of the proximal humerus from an unfractured portion of the humerus [32,33]. 

This SSM could be extrapolated to PHFs because it uses reference points on the unfractured humeral diaphysis. Retroversion, inclination, height, the radius of curvature, and posterior and medial offset were defined based on diaphyseal shape, using reference points in the bicipital groove (Figure 3A). This allowed for a three-dimensional prediction of a pre-fracture humerus “blueprint” (Figure 3B).

3. Anatomical positioning of the pre-fracture proximal humerus

Predictive modeling of the pre-fracture proximal humerus did not always restore the humeral head to an anatomic position with respect to the glenoid (due to humeral shaft injury displacement relative to the glenoid).

**Figure 4:**
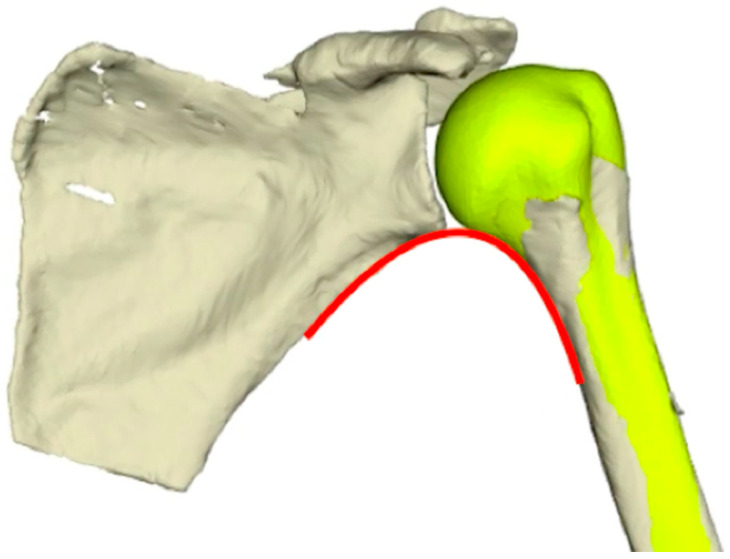
Positioning of the pre-fracture humerus relative to the glenoid. The pre-fracture humerus was translated vertically and axially rotated to restore the glenohumeral joint space and the scapulohumeral arch [34].

To reconstruct the pre-fracture shoulder, the predicted humerus was automatically repositioned opposite the glenoid with 0° of flexion, neutral rotation, and 30° of abduction (position of neutral rotation sling inside the CT scanner). Manual adjustments were also applied to improve the anatomic position to reconstitute the scapulohumeral arch and glenohumeral joint space [34] (Figure 4).

4. Computerized reduction of bone fragments

**Figure 5:**
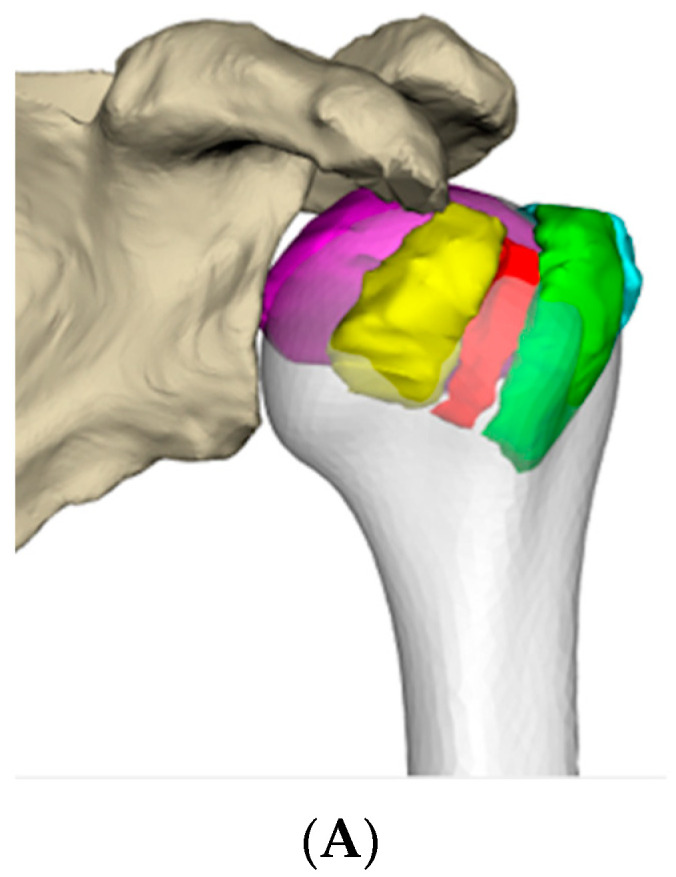

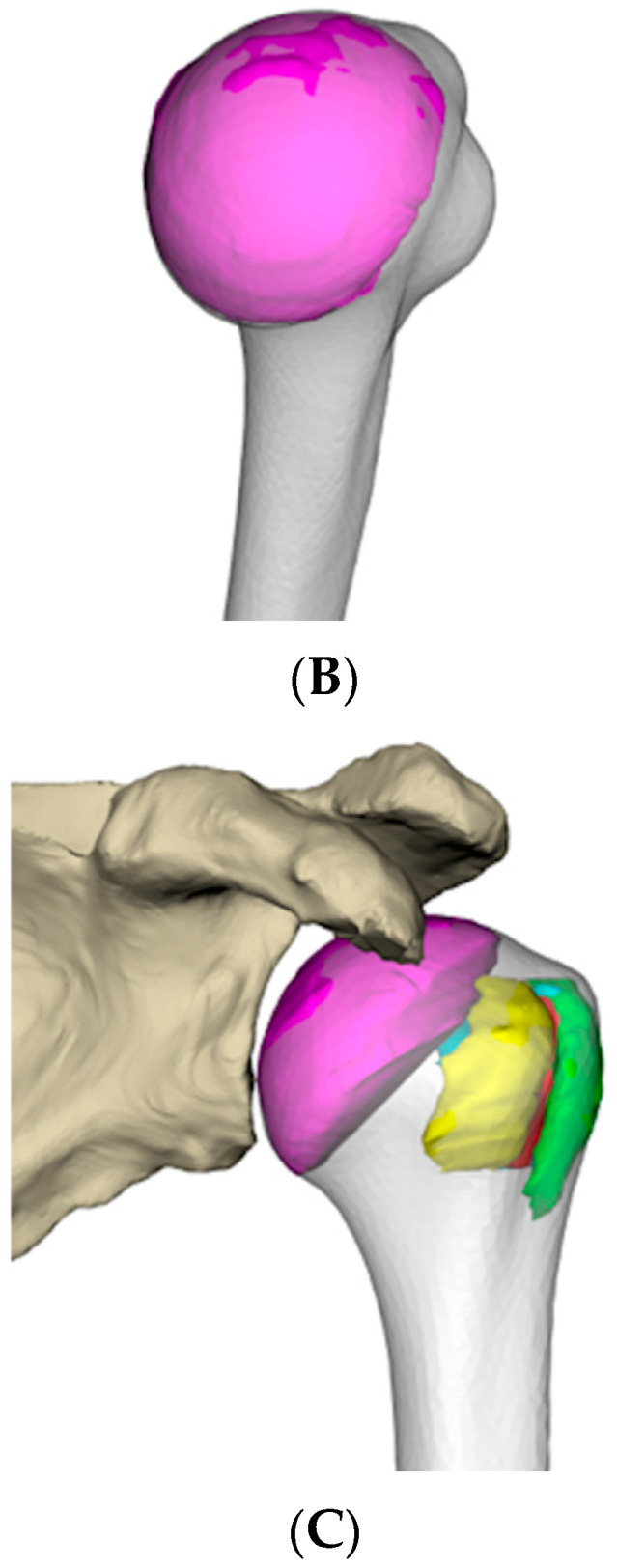
Computerized reduction of fractured bone fragments. (**A**) The 3D mesh of the fracture fragments (purple, yellow, green, and blue) is superimposed onto the predicted pre-fracture proximal humerus (grey). The fragments are not reduced at this stage. (**B**) Each fragment is reduced manually. Here the fractured head (in violet) is reduced based on the pre-fractured humerus (grey). The maneuver is repeated fragment by fragment to obtain an anatomical reduction. (**C**). At the end of this step, the fracture is reduced. The greater tuberosity is green at its anterior part and blue in its posterior part, the lesser tuberosity is yellow. The anatomy of the proximal pre-fracture humerus is restored.

The final step was to reduce the bone fragments to their anatomical position on the pre-fracture proximal humerus blueprint. For this, a specific tool was developed by IMASCAP (Plouzané, France) with the help of a surgical team specialized in shoulder surgery. This tool displays the previously segmented fracture fragments and the predicted pre-fracture proximal humerus layer in the same window (Figure 5A). Each fragment was then manually reduced to its anatomical position on the pre-fractured humerus by an orthopedic surgeon (Figure 5B,C). A rigid three-dimensional transformation matrix was thus generated for each fracture fragment, recording the translations and rotations required to move the fragment from the displaced injury position to the native pre-fracture position.

5. Definition of the 3 humeral reference planes

To obtain translational and rotational displacement values, 3 anatomical reference planes were defined on the pre-fracture humerus (axial, sagittal, and coronal) based on the following algorithm:

1. Diaphyseal axis ($\vec{axial}$): defined by the axis of the humeral diaphysis cylinder, according to the method of least squares;
2. Anatomical neck plane: defined by manual selection of 10 points on the anatomical neck and a plane fit to these points by the least squares method;
3. Anatomical neck axis ($\vec{anatneck}$): the normal (perpendicular) vector to the plane of the anatomical neck, towards the glenoid;
4. Coronal plane axis calculation: $\vec{coro}$ = $\vec{axial}$ ^ $\vec{anatneck}$ (perpendicular to both axial and anatomical neck vectors);
5. Sagittal plane’s axis calculation: $\vec{sag}$ = $\vec{axial}$ ^ $\vec{coro}$ (perpendicular to both axial and coronal vectors).

**Figure 6:**
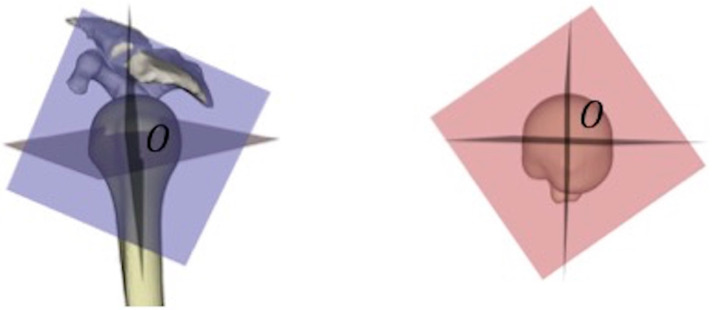
Three anatomical reference planes with the center O.

The marker O was defined as the center of the pre-fracture humeral head (center of the sphere containing the humeral head). Each plane was then defined by its vector originating from the center of the humeral head O (Figure 6).

6. Computerized measurements of displacement

Fragment manual reduction allowed three-dimensional measurement of the translational and rotational displacements of each fragment. 

The path needed to reduce each fragment was represented as a rigid transformation matrix M (4 × 4 matrix, Equation (1)).

(1) $$M=\left(\begin{array}{cccc} a_{11} & a_{12} & a_{13} & a_{14}\\ a_{21} & a_{22} & a_{23} & a_{24}\\ a_{31} & a_{32} & a_{33} & a_{34}\\ 0 & 0 & 0 & 1 \end{array}\right)\;y=Mx$$

On 3D Rigid Transformation Matrix, M represents the rigid transformation matrix containing the translations ($a_{14},\;a_{24}\;and\;a_{34})$ and rotations ($a_{11}\;and\;a_{33}$) required to move each fragment from its original position to its reduced position. Transformations do not include resizing.

This matrix was then decomposed into two components: three translations and three rotations.

### 2.5. Translation Analysis

Each fragment’s displacement was independently measured. The displacement was a function of the location of the centroid of the fragment (defined by the least squares method) before reduction ($G_{P}$) and after reduction ($G_{R}$); these 2 points define the three-dimensional vector ($\vec{GpGr}$) = $\vec{Trans\left(P\right)}$. This vector was projected onto each of the 3 reference planes to obtain a 2D measurement of the translations in the axial, coronal, and sagittal planes. 

The measurement of the absolute three-dimensional translation (distance in 3D space between the centroid of the fragment before and after reduction) was also performed. Its length corresponded to the vector norm $\left\|\vec{Trans\left(P\right)}\right\|=\sqrt{x^{2}+y^{2}+z^{2}}$ (in millimeters).

### 2.6. Rotation Analysis

The rotation of each of the fragments was measured in 3 dimensions, defined by an axis of rotation (unit vector determined from the transformation matrix) and a rotation angle. Because this 3D angle does not correspond to any standard surgical measurement, two-dimensional measurements were also performed, in the coronal (varus/valgus), sagittal (anterior-posterior tilt), and axial (anteversion–retroversion) planes, as is typical for PHFs.

### 2.7. Comparison to Neer’s Criteria

Finally, we compared the computerized measurements of $\vec{Trans\left(P\right)}$ and coronal, sagittal, and axial rotations to Neer’s reference thresholds of 10 mm and 45° displacements. 

### 2.8. Statistical Analyses

Discrete outcomes were reported as absolute and relative frequencies, while continuous outcomes were reported as means with 95% confidence intervals. The normality of data was assessed with the Shapiro–Wilk test. 2D and 3D measurements were compared with a two-tailed paired Student t-test with a significance level of 0.05. 

Inter-observer agreement was determined with the use of absolute percentages of agreement and unweighted Fleiss’ kappa values. Fleiss’ kappa values were interpreted according to Landis and Koch [35].

Data collection and statistical analyses were performed with EasyMedStat (www.easymedstat.com (accessed on 25 September 2019); v3.9; Levallois—Perret; France).

## 3. Results

Independent analysis by the 3 observers categorized fractures as “displaced” in 51/77 cases (66%), and classified them as 1-part (*n* = 26), 2-part (*n* = 24), 3-part (*n* = 20), or 4-part (*n* = 7).

There was a moderate agreement between observers (κ = 0.47; CI [0.40; 0.55]).

### 3.1. Computerized Fracture Description

Three-dimensional computed measurements of epiphyseal fragments in our series showed that 47% of PHFs (36/77) were displaced according to at least one of Neer’s criteria.

### 3.2. Humeral Head Rotation

**Figure 7:**
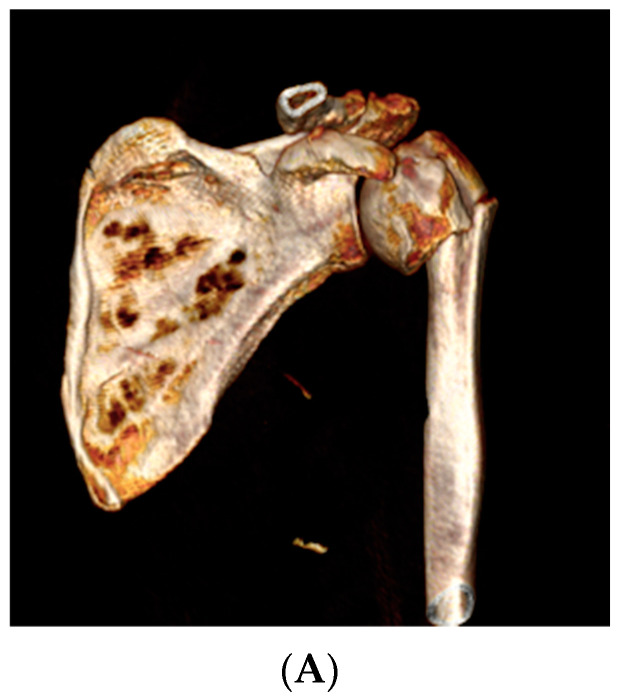

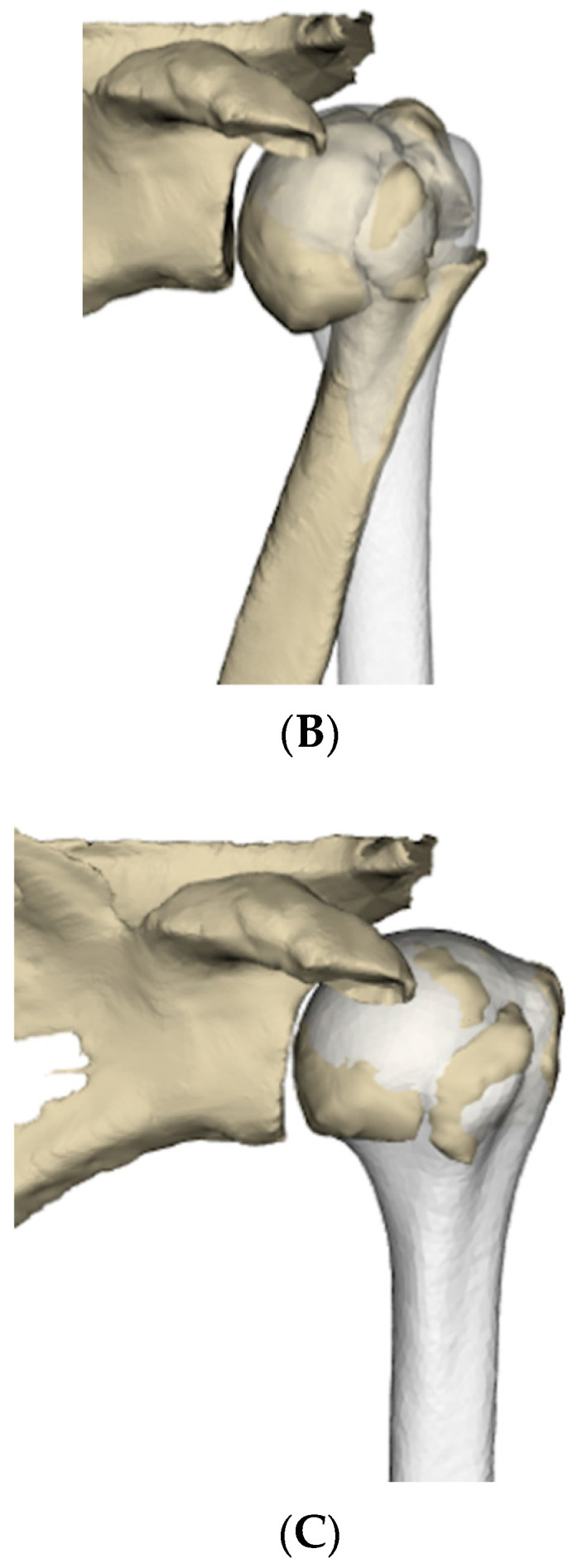
Example of rotation in the coronal plane (varus). (**A**) Surgical neck fracture (3D scanner); (**B**) In white, the proximal pre-fracture humerus is positioned in a neutral anatomic position at the glenoid. In beige, the fractured humerus with the diaphysis displaced in adduction and the head displaced in varus. (**C**) The fractured humerus is reduced onto the pre-fracture humerus. The computerized measurement indicates a 33° varus displacement.

**Figure 8:**
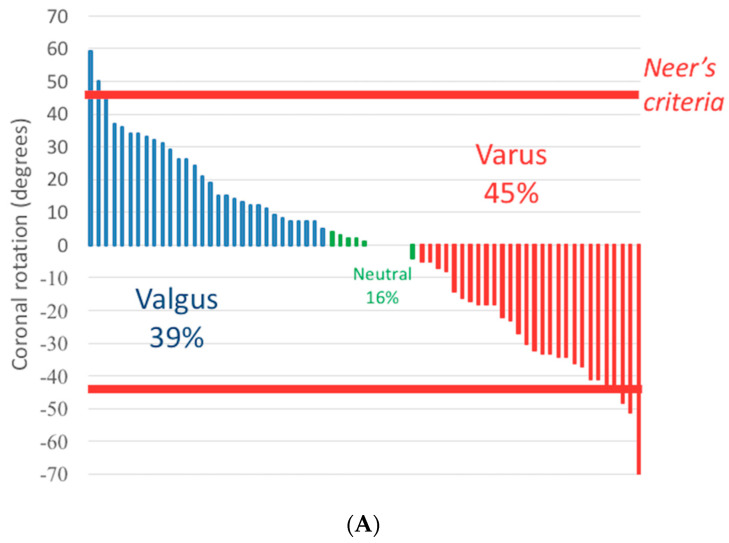

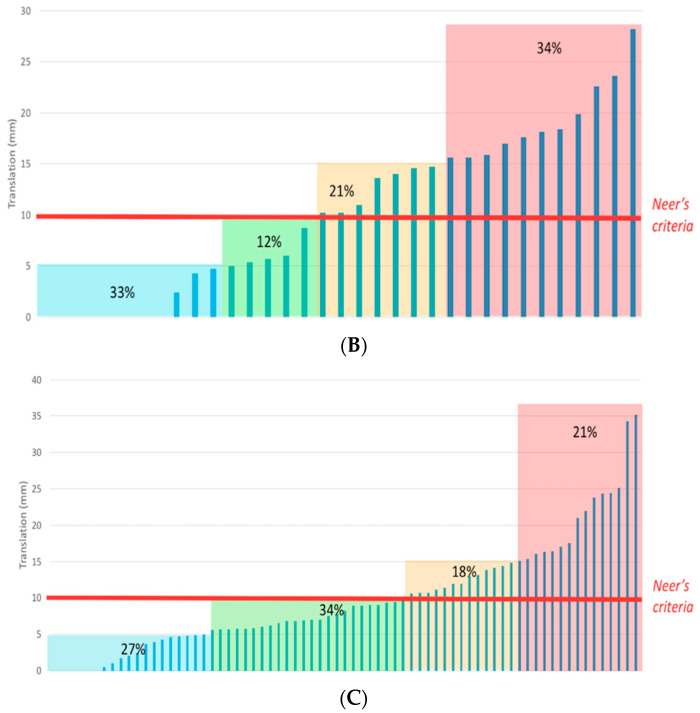
Measurement of rotations and translations by the software. (**A**) Coronal rotation of the head in the varus and valgus measured by our tool; 8% of fractures met Neer’s criteria. (**B**) Three-dimensional translation of greater tuberosity measured by our tool. 39% of the fractures met Neer’s criteria. (**C**) Three-dimensional translation of the lesser tuberosity measured by our tool. 53% of the fractures met Neer’s criteria. (**B**,**C**) The red line indicates Neer’s criteria threshold. Tuberosity translations are classified as <5 mm (light blue), 5−9 mm (green), 10−15 mm (orange), and >15 mm (red).

The surgical neck was fractured in 90% of PHFs (69/77) (Figure 7). In fractures of the surgical neck, most rotational displacements (37/69) measured between 10° and 40° in the coronal plane. We observed 39% of fractures displaced in the valgus by 5° or more, 45% displaced in the varus by 5° or more, and 16% with less than 5° coronal rotation (Figure 8). In 8% of cases, displacements were greater than Neer’s threshold of 45°.

Valgus-displaced fractures were associated with posterior rotation greater than 5° (in the axial plane) in 48% of cases (13/27), and with an anterior rotation greater than 5° in 11% of cases (3/27). Varus-displaced fractures were associated with a retroversion greater than 5° in 61% of cases (19/31), and with an anteversion greater than 5° in 19% of cases (6/31) (Table 2). Overall, 13% of fractures (10/77) exhibited posterior rotation of the humeral head fragment exceeding 45° (2 valgus, 8 varus), but we did not observe any fractures with anterior rotation greater than 45°.

### 3.3. Tuberosity Translation

The greater tuberosity was fractured in 91% of PHFs (70/77), but the displacement was below Neer’s 10 mm threshold in 61% of cases (43/70). The lesser tuberosity was fractured in 44% of PHFs (34/77), while displacement was below Neer’s criteria in 47% of cases (16/34) (Figure 8).

These displacements corresponded to translations in 3D space (Figure 7 and Figure 8).

This measure in 3D was projected on 2D planes to calculate a 2D measurement, as would be done on multiplanar reconstructions. These 2D measurements were compared to the 3D measurements. The 2D projections were found to underestimate the 3D displacement of the fragment by an average of 2.21 mm (CI_95%_ [1.618; 2.805], range: 0 to 34.7) in the coronal plane, 2.6 mm (CI_95%_ [1.578; 3.785], range: 0 to 9.3) in the sagittal plane, and 1 mm (CI_95%_ [0.636; 1.398], range: 0 to 34.9) in the axial plane (*p* < 0.001 for all measurements).

### 3.4. Accuracy of Manual Measurements

Using software measurements as a reference control, the three observers who participated in this study correctly described the degree of varus/valgus displacement in only 35% of cases. For the greater tuberosity (*n* = 70), again using the software as a reference control, observers accurately categorized fragment translation in 61% of cases (<5 mm, 5–9 mm, 10–15 mm, or >15 mm). For the lesser tuberosity (*n* = 34), this accuracy was 49%.

## 4. Discussion

The decision to operate and selection of appropriate surgical modality for PHF treatment is largely based on bone fragment displacement and more specifically tuberosity displacement [6,7]. Rather than evaluating Neer’s conventional criteria, the purpose of the present study was to more accurately measure 3D displacements of epiphyseal fracture fragments using proximal humerus pre-fracture anatomy prediction (statistical shape modeling, SSM). Additionally, we endeavored to compare these novel 3D computational measurements with those obtained using previously described manual 2D methods [3]. While Neer’s criteria [3] were defined somewhat arbitrarily based on available measurements in 2D, one can imagine that a rotation of 20° in two planes, for example, can have more important clinical consequences than a single plane rotation of 45°. Accordingly, our goal was to perform a retrospective feasibility study as a first step to refining proximal humerus fracture displacement measurements using 3D modern computational tools.

Using standardized CT studies of 77 fractured shoulders, our 3D computer measurements showed that 47% of PHFs (36/77) were displaced according to Neer’s criteria (>10 mm or >45°). Data analysis confirmed that a computerized method could allow 3D measurements of fracture displacements in a manner significantly more accurate compared to traditional manual 2D measurements. We found that 2D methods consistently underestimate the displacement of the tuberosity fragments and cannot measure rotational displacements. Although manual measurement of fragment displacement is time-consuming, it is a prerequisite to validate the future development of automated or semi-automated methods. With knowledge of the 3D bone fragment displacements in PHFs, we aim to empower surgeons to preoperatively anticipate the reorientation and repositioning required for the reduction of these fragments, as well as select the optimal method of fixation and reconstruction. The clinical relevance of millimeter displacement measurements remains unknown, but we believe that it is important to have a measurement that is as reproducible as possible and that can be used as a gold standard.

The presented method standardizes the three-dimensional measurement of translational and rotational displacements of the 3 epiphyseal fracture fragments in PHFs. Our methodology is original and robust, based on the use of a predicted pre-fracture humerus “blueprint” that serves as a layer onto which displaced fragments are computationally reduced. This enables the quantification of fragment displacement even in cases of complex fractures where no fixed reference can be used. Our computerized measurement algorithm enables the characterization of fragment displacements in 3D space, which cannot be done with traditional measurements on X-rays or CT scans. Even when using 3D reconstructions of the humerus from CT scans, every measurement is traditionally performed on a 2D projection, whereas our method quantifies 3D distances.

Previously, Russo and al. performed a morphovolumetric study [36] of head malposition in PHF with a new classification [37]. This morphovolumetric study [36] investigated head displacement in relation to the shaft and bone loss, but only in impacted PHFs. Furthermore, tuberosity displacements were not explicitly measured, despite the paramount importance of this fracture characteristic [6,28].

SSMs have already been used in other studies [33] and for other joints, in particular the wrist [38] and diaphyseal and proximal femur fractures [39,40]. In these prior works the statistical model was used for reduction and osteosynthesis analysis to guide surgeons, but did not allow evaluation or measurement of displacement. Only one prior study examined the computerized reduction of PHF. In [41], the authors used a semi-automated method to reconstruct 12 humeri by aligning the fracture fragments edge-to-edge using an algorithm, but excluded fragments with a surface area of less than 195 mm^2^. Finally, another group used machine learning to automatically classify PHFs according to Neer’s classification based on standard AP view X-rays, with a reported accuracy between 65% and 86% [42] but did not report displacements.

**Figure 9:**
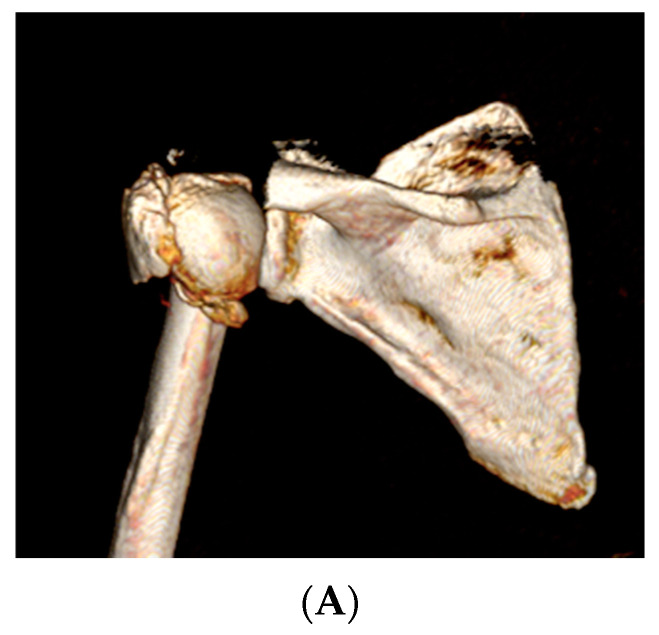

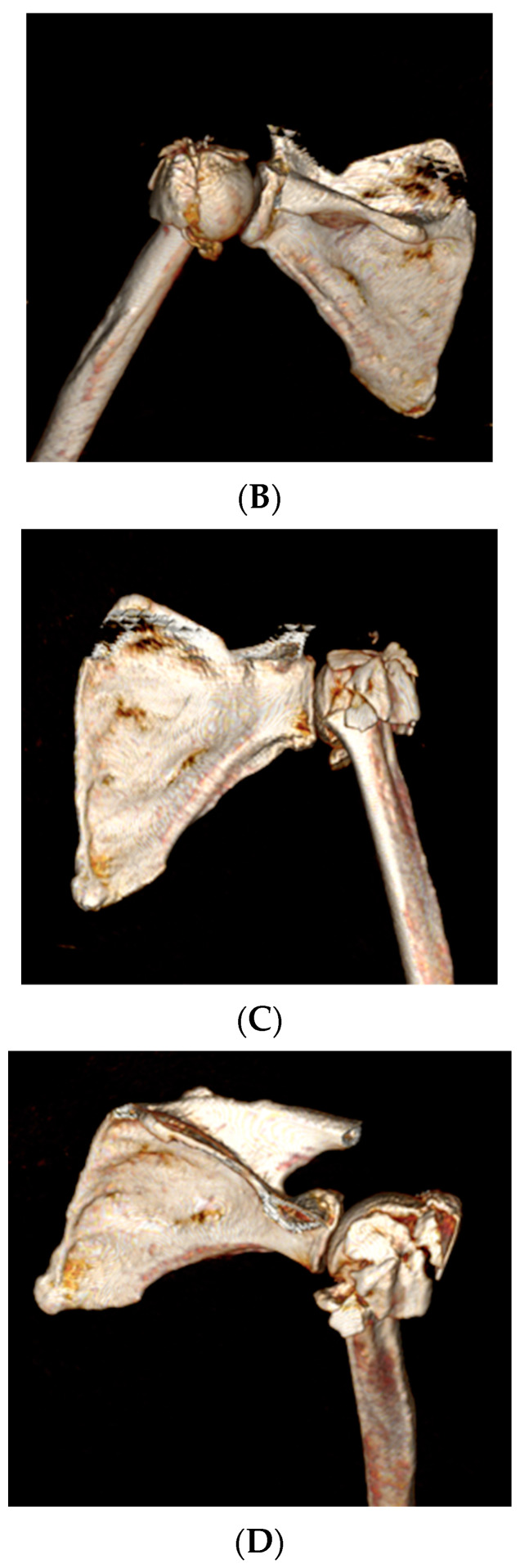
Example of a varus displacement with associated humeral head rotation from different perspectives. (**A**) AP view; (**B**) oblique anterior view; (**C**) oblique posterior view; (**D**) posterior view.

Overall, the paucity of prior work and our own findings confirm that the three-dimensional nature of PHF displacements makes 2D analysis difficult. For instance, we observed that coronal rotation is frequently accompanied by sagittal (anterior-posterior) and axial (anteversion-retroversion) rotations (Table 2). This fact reduces the ability of the observer to appreciate varus/valgus rotations, which inherently will depend on the perspective from which the fracture is observed (Figure 9).

It can be readily appreciated that—depending on the observer’s perspective relative to the shoulder in 3D space—the extent of varus and rotational displacements appears to be quite different.

Our method has several limitations. In the first segmentation step, some small fragments were excluded and only the 4 primary fragments were considered (as historically described in the PHFs [3,43]). However, our technique minimizes the impact of these missing fragments as we did not attempt to reduce fragments edge-to-edge (as has been proposed previously [41]). Additionally, there may be errors in the pre-fracture proximal humerus prediction originating from the SSM used. These average errors and standard deviations were estimated by Poltaretskyi et al. [32] for retroversion, inclination, height, radius of curvature, posterior offset, and medial offset: 3.8° (±2.9°), 3.9° (±3.4°), 2.4 mm (±1.9 mm), 1.3 mm (±0.9 mm), 0.8 mm (±0.5 mm), and 0.9 mm (±0.6 mm), respectively. Interestingly, these prediction errors actually appear to be smaller for certain parameters compared to those generated when using the contralateral humerus as a control [44]. Another potential source of error is the position of the predicted pre-fracture humerus, which relies on subjective assessment of the scapulohumeral arch and glenohumeral joint restoration. Additionally, we did not analyze the displacement of the diaphysis, which requires different methodologies and deserves further investigation in the future. Finally, the analysis protocol was time-consuming. It took approximately one hour for the surgeon and thirty minutes for the engineer (per fracture) to carry out measurements. Many steps, if not the entire procedure, will need to be automated before this process can be used in everyday practice. Analysis of diaphyseal displacement and improving generalizability of the method to additional bones and fractures will be the subject of future work. This study is also limited by the probable under-representation of minimally displaced fracture patterns, as CT scans are less frequently requested for these cases. We did not perform inter-observer or intra-observer analyses for the computational methods as we plan to automate this process and eliminate the source of variability. Finally, for 3 excluded cases our methods failed to predict the pre-fracture proximal humerus, representing 3.75% of cases. In these cases, the software was not able to generate an SSM from the unfractured shaft due to a defect in the CT scan images.

We believe this method is a promising tool to improve the quantification and standardization of fracture descriptions when treating PHFs and potentially other types of fractures. An approach that incorporates the prediction of the pre-fracture humerus boasts the advantage of not requiring a CT scan of the contralateral shoulder [45,46,47], thus limiting patient radiation, and is feasible even in cases with a contralateral shoulder injury, prior surgery, or fracture sequelae. Displacements have been measured in three dimensions, with a mathematically defined and precise method. We included a wide spectrum of fractures, ranging from non-displaced to highly displaced fractures, which allowed us to develop our tool step-by-step, calibrating measurements based on cases with little or no displacement. Finally, we carried out this work in collaboration with engineers from a company specialized in the field of 3D imaging of the shoulder and surgical applications [48,49].

## 5. Conclusions

The results of this investigation confirm the feasibility of precisely measuring the three-dimensional displacement of fractured epiphyseal proximal humerus bone fragments using a computerized measurement method. Development of this 3D tool lays the foundation for improving our ability to quantify and standardize the description of complex fracture morphology and eventually automate the diagnosis and classification of PHFs. Ultimately, this is an important first step in improving the fidelity of patient-specific data for fractures managed both operatively and non-operatively, and eventually refining best practices for therapeutic decision-making.

## Figures and Tables

**Table 1:** Epidemiology.

Variable	Average (Minimum–Maximum) Frequency (%)
Age	71 years (29–95)
Female gender	63 (81%)
Laterality	Right: 41 (53%)/Left: 36 (47%)
Dominant side	39/77 (51%)
Mechanism	Ground level fall: 61 (79%)
	Fall from height: 3 (4%)
	Motor vehicle accident: 8 (10%)
	Sports accident: 5 (7%)

**Table 2:** Axial (anteversion/retroversion) and sagittal (anterior tilt/posterior tilt) rotations according to the coronal (varus/valgus) rotation.

		Coronal Rotation					Total
		Valgus > 45°	Valgus 5–45°	<5°	Varus 5–45°	Varus > 45°	
Axial rotation	Retroversion > 45°	0	2	0	6	2	10
	Retroversion 5–45°	0	11	1	10	1	23
	<5°	1	10	16	6	0	33
	Anteversion 5–45°	2	1	2	6	0	11
	Anteversion > 45°	0	0	0	0	0	0
Sagittal rotation	Posterior tilt > 45°	0	0	0	3	1	4
	Posterior tilt 5–45°	1	17	3	17	1	39
	<5°	2	6	15	3	0	26
	Anterior tilt 5–45°	0	1	1	5	0	7
	Anterior tilt > 45°	0	0	0	0	1	1
Total		3	24	19	28	3	77

## Data Availability

Restrictions apply to the availability of these data. Data were obtained from the company IMASCAP (Plouzané, France) and may be available upon reasonable request from the authors with the permission of IMASCAP.
